# Prevalence and Factors Associated with Burnout among Community Pharmacists in Saudi Arabia: Findings and Implications

**DOI:** 10.3390/healthcare12181834

**Published:** 2024-09-13

**Authors:** Ibrahim S. Alhomoud, Alian A. Alrasheedy

**Affiliations:** Department of Pharmacy Practice, College of Pharmacy, Qassim University, Qassim 51452, Saudi Arabia; i.alhomoud@qu.edu.sa

**Keywords:** burnout, community pharmacists, policy, quality of life, resilience, stress, well-being

## Abstract

Burnout has negative consequences for the well-being of healthcare professionals and for the safety of patients. The prevalence of burnout varies among health professions and practice settings and across studies. Consequently, this cross-sectional study aimed to determine the prevalence and factors associated with burnout among community pharmacists in the Qassim region, Saudi Arabia. The study used the Copenhagen Burnout Inventory (CBI) to assess the burnout. The CBI consists of three scales, including personal burnout, work-related burnout, and client-related burnout. A total of 226 community pharmacists participated in the study. Of these, 63.72% were male, and 44.25% had experience of 1–5 years as community pharmacists. The prevalence of personal burnout was 83.63%, while the prevalence of work-related burnout was 83.19%, and the prevalence of client-related burnout was 76.11%. The prevalence of burnout was higher among younger age, early-career pharmacists; staff pharmacists; pharmacists working ≥6 days per week; and those working in pharmacies with fewer pharmacy teams. Multivariable logistic regression showed that compared to five workdays, working six and seven days per week was an independent risk factor for developing personal burnout [(adjusted odds ratio (aOR) = 3.60: CI = (1.29–10.05), *p* = 0.014) and (aOR = 4.72: CI = (1.17–19.08), *p* = 0.030), respectively]. Similarly, pharmacists working in pharmacies with one or two pharmacists were at higher odds of developing personal burnout compared to others working in a larger team (aOR = 3.41: CI = (1.09–10.66), *p* = 0.035). For work-related burnout, working six and seven days per week was also an independent risk factor [(aOR = 5.78: CI = (1.96–17.06), *p* = 0.001), and (aOR = 8.38: CI = (1.99–35.27), *p* = 0.004, respectively)]. For client-related burnout, staff pharmacists were at higher odds of developing client-related burnout compared to pharmacy managers [(aOR = 2.28: CI = (1.01–5.14), *p* = 0.046)]. Overall, the prevalence of burnout is alarmingly high among community pharmacists in Saudi Arabia. Consequently, it is crucial to urgently address it through robust initiatives, strategies, and interventions that support the well-being, quality of life, and resilience of community pharmacists.

## 1. Introduction 

Burnout is an occupational phenomenon that develops due to prolonged exposure to work-related stressors while lacking adequate resources and support to efficiently cope with this stress in the workplace [1,2,3]. In 2019, in the 11th revision of the International Classification of Diseases (ICD-11), burnout was defined as “a syndrome conceptualized as resulting from chronic workplace stress that has not been successfully managed” [4]. It is characterized by three dimensions, including emotional exhaustion (i.e., feelings of energy depletion, fatigue, tiredness, and weariness), cynicism or depersonalization (i.e., disconnection from work or unconcern towards the one’s job and/or the clients or patients receiving the service, feelings of negativity), and reduced professional efficacy and accomplishments [2,4]. The term “burnout” was first described in relation to healthcare workers by Herbert Freudenberger in the middle of the 1970s [5]. Since then, the recognition of burnout within the healthcare profession has gradually increased globally [6,7,8,9,10,11,12]. Moreover, burnout has become a more pronounced concern recently during the COVID-19 pandemic. Recent studies have consistently shown a high prevalence of burnout among healthcare workers globally and in Saudi Arabia [13,14,15,16,17,18,19,20,21,22,23]. For example, a study that included 646 healthcare workers in Saudi Arabia found a high burnout rate of 75%. The study indicated that notable contributing factors to burnout included age, years of experience, increased working hours, mental stress, and exposure to COVID-19 [23]. However, the study did not specifically address the burnout rate among community pharmacists.

The prevalence of burnout varies among different healthcare sectors, practice settings, and among studies in the literature. A recent scoping review showed large variations in the prevalence of burnout among healthcare professionals (ranging from 4.3 to 90.4%) [24]. Another systematic review reported a prevalence among European physicians ranging from 2.5 to 72% [25]. Among pharmacists, the burnout prevalence was estimated to be 51% [26] and higher than 80% and 90% among community pharmacists according to some studies [27,28]. The variations in the reported prevalence depend on several factors, including the country of the study, demographic characteristics (e.g., age, occupation, educational level), psychological conditions (e.g., stress, coping strategy, anxiety), social factors (e.g., family life), work organization (e.g., workload, support, staff and resources, work environment), and other factors (e.g., dealing with COVID-19 cases) [24]. 

Pharmacists are particularly susceptible to burnout due to the unique demands of their profession. Burnout can affect pharmacists at any career stage and is prevalent across various pharmacy sectors [26,29,30,31,32,33,34]. The current research indicates that burnout is notably high among community pharmacists [27,28,35,36,37,38]. The higher prevalence of burnout among pharmacist is likely due to working extended hours, the pace of the work environment, high volumes of patients and prescriptions, poor work/life balance, and excessive workload [26,28,36,38]. Additionally, community pharmacists face increased work demands due to their high accessibility to the public [39]. Other factors contributing to burnout included lack of sufficient resources to cope with work-related stressors [37], the need for more resilience support [37], lack of opportunity to make choices, and insufficient financial rewards [38]. The impact of burnout extends beyond the individual clinician to affect patient safety [40,41]. A systematic review revealed that high levels of burnout among healthcare professionals, including pharmacists, are associated with increased medical errors and compromised patient safety [41]. Consequently, it is crucial to conduct studies and investigations focusing on detailed assessments of burnout prevalence and developing strategies for mitigation within the healthcare sector, especially among community pharmacists. This is given that recent studies indicated that burnout among community pharmacists remains insufficiently explored, highlighting the need for more research in this sector. This includes more research to generate data and evidence to support interventions and strategies that are specific to the unique practice setting of community pharmacists [36,38].

To our knowledge and based on extensive search in databases and search engines including Google Scholar, PubMed, Scopus, and the Web of Science, there is a paucity of research on burnout among community pharmacists in Saudi Arabia. Consequently, as the prevalence of burnout varies among different healthcare sectors, practice settings, and among studies, and given the gap in the literature on the prevalence and the factors associated with burnout among community pharmacists in Saudi Arabia, this study would provide valuable insights and a timely contribution to this topic. This is particularly important because the roles of community pharmacists in Saudi Arabia have been recently expanded to include a variety of new clinical and non-clinical activities (e.g., transfer of pharmaceutical services and dispensing of medicines from the public primary healthcare centers and some hospitals to community pharmacies via the e-prescribing service) [42]. Moreover, the community pharmacy sector witnessed substantial growth in recent years in Saudi Arabia [43,44]. From 2007 to 2022, the number of community pharmacists increased substantially by 98.02%. The recent statistics in 2022 showed that there were 21,648 community pharmacists practicing in a total of 10,347 community pharmacies across 13 administrative regions in Saudi Arabia. This represented approximately 63.6% of the pharmacy workforce in the healthcare sector [44]. Consequently, this study aims to address this topic and fill the gap, particularly as there are limited studies on burnout and the well-being of community pharmacists globally [36]. Therefore, this study can provide valuable data and insights into burnout among community pharmacists and provide recommendations for health policy to mitigate burnout risks within this sector.

## 2. Methods 

### 2.1. Study Design, Setting, and Population 

This is a cross-sectional, questionnaire-based study conducted with community pharmacists practicing in the Qassim region, Saudi Arabia. The study was conducted over three months (April to June 2024). 

### 2.2. The Sample Size and Sampling Method 

According to recent data, there were 498 community pharmacies in the Qassim region in 2022. The number of community pharmacists is roughly estimated to be 1100 [43,44]. Consequently, with a margin error of 5%, confidence level of 95%, and variability level of 50%, the sample size is estimated to be 285 community pharmacists. The variability level of 50% was used to determine the sample size as the response distribution of the variable of interest (i.e., prevalence of burnout) is not known. Consequently, this yielded a more conservative sample (i.e., largest sample size) [45]. The sample size calculation was performed using the Raosoft^®^ sample size calculator (http://www.raosoft.com/samplesize.html, accessed on 5 February 2024) [45]. The convenience sampling method was used in this study, as we approached community pharmacists across the region based on the geographical convenience of the data collectors. 

### 2.3. The Study Instrument/Questionnaire

The questionnaire used in this study consisted of two sections. The first section was related to the participants’ demographic data and work-related characteristics. It consisted of 12 items, including age, sex, years of practice experience as community pharmacists, current position in the pharmacy, highest degree earned, marital status, number of working hours per day, number of working days per week, number of pharmacists employed in the pharmacy, number of patients and customers served per day, number of learners trained in the pharmacy, and assignment to night shift in the pharmacy. The second section of the questionnaire included the Copenhagen Burnout Inventory (CBI). CBI was used in this study because it is a well-validated instrument and a widely used tool to measure burnout and is available in the public domain. This allowed our study to build on the existing body of evidence and provides a basis for comparisons with the findings of other studies [46,47]. The CBI consists of 19 questions divided into three subdimensions/scales (i.e., three burnout scales). These include the personal burnout scale (six questions), the work-related burnout scale (seven questions), and the client-related burnout scale (six questions). The personal burnout questions assess how often participants feel tired, physically and emotionally exhausted, unable to cope, worn out, and susceptible to illness. The work-related burnout questions evaluate emotional exhaustion and burnout from work, frustration with work, and physical exhaustion at the end of the workday. Additionally, the work-related burnout questions assess whether the respondent feels exhausted at the thought of another day of work, finds each working hour tiring, and has enough energy for family and friends during leisure time. The client-related burnout questions evaluate whether working with clients is challenging or frustrating, drains energy, and makes respondents feel they give more than they receive, as well as whether they are tired of this work and question their ability to continue [47].

The CBI is a valid instrument with high reliability and has good psychometric properties to measure burnout. It has been used in many cultures and countries [46,48] and was used to measure burnout among healthcare professionals and clinicians [49,50,51,52,53,54,55], including pharmacists [28,56,57]. Twelve questions have frequency responses on a five-point scale: always (100), often (75), sometimes (50), seldom (25), never/almost never (0). Seven questions have five response options: to a very high degree (100), to a high degree (75), somewhat (50), to a low degree (25), to a very low degree (0). The total score of the scale is the average of the scores of the questions in the scale. The scores range from 0 to 100 points, with a higher score indicate a higher level of burnout [46,47]. A total score of ≥50% is used as a cut-off point for burnout for each scale. Furthermore, burnout is classified according to its severity, with scores less than 50 indicating low/no burnout, 50–74 indicating moderate burnout, 75–99 indicating high burnout, and 100 indicating severe burnout [28,58,59]. 

Prior to administration of the questionnaire, it was administered to five community pharmacists practicing in the Qassim region to ensure its applicability, suitability, comprehension, and clarity to the target population. Their feedback was positive and indicated that the questionnaire was easily understandable, clear, and relevant to their practice setting. The reliability of CBI scales in this study was assessed. The Cronbach’s alphas for all the three scales were high (personal burnout, α = 0.917; work-related burnout, α = 0.814; client-related burnout, α = 0.910). 

### 2.4. Data Collection Procedure and Administration of the Questionnaire 

Google Forms was used to create the online survey and collect the data. A link to access the survey was generated, and an invitation letter was prepared. The participants were invited to participate in this study through in-person field visits to the community pharmacies by the researchers. In addition, a link to the survey was sent via the WhatsApp^®^ application (https://www.whatsapp.com, accessed on 1 April 2024) to the contact numbers of community pharmacists in the contact network of the researchers. Before the survey was administered, participants were informed of the study’s aims, confidentiality, and voluntary participation. In addition, the participants were informed that the data were collected anonymously through the online survey and that no information that could identify the participants was collected. The completion of the survey and submitting the response online by participants were deemed to be a consent to participate. In this study, the Google Forms platform was set up to require participants to answer all questions before submitting their responses. This ensured that every response was complete, eliminating the issue of missing data.

### 2.5. Data Analysis 

Descriptive statistics were used to summarize the data, including frequencies, percentages, and mean and standard deviation (SD). Inferential statistics, including the chi-squared test and the equivalent Fisher’s exact test, were used to examine the association between the dependent/outcome variable (i.e., burnout) and the independent variables (i.e., demographic and work-related factors). Variables with *p* < 0.1 in the univariable analysis were included in the multivariable logistic regression to determine the independent factors associated with developing burnout [60,61,62]. The results are reported as odds ratios (ORs) and 95% confidence intervals (CIs). The significance level was set at a two-sided *p* < 0.05. Statistical analyses were performed using IBM SPSS statistics for Windows, version 20.0.

### 2.6. Ethical Approval 

The study protocol was approved by the Regional Research Ethics Committee, Qassim region, Saudi Arabia (Approval No. 607-45-013353). The study was carried out in accordance with the ethical principles of the World Medical Association’s Declaration of Helsinki. 

## 3. Results

### 3.1. Participants’ Demographic Data and Work-Related Characteristics 

A total of 226 community pharmacists participated in the study (response rate = 79.30%). Out of these, 144 (63.72%) were male, and 82 (36.28%) were female participants. The majority of the participants (n = 151; 66.81%) were in the age group of 23–30 years, followed by 43 (19.03%) in the age group of 31–35 years old, and the remaining participants (n = 32; 14.16%) were ≥36 years old. The participants had varying years of experience as community pharmacists in Saudi Arabia, with 44.25% of participants (n = 100) possessing 1–5 years of experience. Regarding their current position in the pharmacy, 171 (75.66%) were staff pharmacists, and 55 (24.34%) were pharmacy managers. Most of the participants (n = 218; 96.46%) hold the entry-to-practice professional degree (i.e., bachelor’s degree or PharmD) as their highest degree. Regarding the pharmacists’ number of working hours per day, it ranged from less than 8 h (n = 7; 3.10%) to ≥12 h (n = 28; 12.39%), with the largest proportion (n = 93; 41.15%) working 8 h per day, followed by 9 h (n = 47; 20.80%) and 10 h (n = 34; 15.04%). The majority of participants (n = 143; 63.27%) were working 6 days per week. More than half of the pharmacies (n = 126; 55.75%) employed one or two pharmacists. The majority of the pharmacists (n = 176; 77.88%) had assignments to night shift and duties in the pharmacy in the last 12 months. The details of participants’ demographic data and work-related characteristics are presented in Table 1. 

### 3.2. Prevalence and Severity of Burnout among the Study Participants

The prevalence of personal burnout among community pharmacists participating in this study was 83.63%. Out of these, 28.32% were classified as moderate, 41.59% as high, and 13.72% as severe personal burnout. The work-related burnout prevalence was 83.19% among the study participants. Out of these, 35.40% were classified as moderate, 39.82% as high, and 7.96% as severe work-related burnout. The prevalence of client-related burnout was 76.11%. Out of these, 38.50% were classified as moderate, 26.99% as high, and 10.62% as severe client-related burnout. The results are summarized in Table 2. 

### 3.3. Association between Burnout and Participants’ Demographic and Work-Related Characteristics 

The association between the burnout and participants’ demographic and work-related characteristics were examined in this study. Regarding personal burnout, there were statistically significant associations between burnout and participants’ age, the pharmacist’s number of working days per week, and the number of pharmacists employed in the pharmacy (*p* < 0.05). The participants in the younger age groups (of 23–30 and 31–35 years) had a relatively higher prevalence of personal burnout compared to the participants in the age group ≥36 (86.75%, 83.72%, and 68.75%, respectively, *p* = 0.044). The participants working 5 days per week had a lower prevalence (62.50%) of burnout compared to those working 6 days (85.31%) and 7 days per week (88.14%) (*p* = 0.011). Similarly, participants working in community pharmacies with a smaller number of employed pharmacists had a higher prevalence of personal burnout (88.89% for pharmacies with one or two pharmacists) compared with pharmacies with a larger number of pharmacists (68.00% for pharmacies with > six pharmacists) (*p* = 0.001). 

Regarding work-related burnout, there were statistically significant associations between work-related burnout and participants’ age, the years of practice experience as community pharmacists, the position in the pharmacy, the pharmacist’s number of working days per week, and the number of pharmacists employed in the pharmacy (*p* < 0.05). The participants in the younger age groups (23–30 and 31–35 years of age) had a relatively higher prevalence of work-related burnout compared to the participants in the age group ≥36 (87.42%, 81.40%, and 65.63%, respectively, *p* = 0.011). The participants with fewer years of experience had a higher level of burnout compared to those participants with more years of experience (e.g., 88.00% for those with 1–5 years of experience compared to 64.29% for those with >10 years of experience) (*p* = 0.004). In terms of their position, staff pharmacists had a higher prevalence of burnout compared to pharmacy managers (87.13% versus 70.91%, *p* = 0.005). The participants working 5 days per week had a lower prevalence (58.33%) of burnout compared to those working 6 days (86.01%) and 7 days per week (86.44%) (*p* = 0.003). Similarly, participants working in community pharmacies with a lower number of employed pharmacists had a higher prevalence of personal burnout (88.10% for pharmacies with one or two pharmacists) compared with pharmacies with a larger number of pharmacists (76.00% for pharmacies with > six pharmacists) (*p* = 0.008). 

Similarly, there were statistically significant associations between client-related burnout and participants’ age, the years of practice experience as community pharmacists, the position in the pharmacy, and the number of pharmacists employed in the pharmacy (*p* < 0.05). In addition, there was a statistically significant association between client-related burnout and the degree of education, with PharmD pharmacists reporting relatively higher client-related burnout (84.68%) compared to others (i.e., 69.16% for those with a bachelor’s degree, and 50% for pharmacists with a postgraduate qualification) (*p* = 0.006). The results are presented in Table 3.

### 3.4. Associated Factors with Burnout among the Participants

Univariable logistic regression analyses were conducted to explore potential factors associated with burnout (Supplementary Tables S1–S3). Then, multivariable logistic regressions were conducted to determine the independent risk factors associated with burnout among the community pharmacists for personal, work-related, and client-related burnout. For the personal burnout, the logistic regression model showed that the number of working days per week and the number of pharmacists employed in the pharmacy are independently associated with personal burnout. Compared to community pharmacists working 5 days, both pharmacists who work for 6 days and those who work for 7 days are at higher odds of developing burnout [(aOR = 3.60: CI = (1.29–10.05), *p* = 0.014) and (aOR = 4.72: CI = (1.17–19.08), *p* = 0.030), respectively]. Similarly, pharmacists working in pharmacies with one or two pharmacists only are at higher odds of developing personal burnout compared to pharmacies with more pharmacists (aOR = 3.41: CI = (1.09–10.66), *p* = 0.035).

For work-related burnout, the logistic regression analysis showed that the pharmacists’ number of working days per week is independently associated with work-related burnout, with pharmacists working 6 days per week (aOR = 5.78: CI = (1.96–17.06), *p* = 0.001) and those working 7 days per week (aOR = 8.38: CI = (1.99–35.27), *p* = 0.004) at higher risk of developing work-related burnout compared to those pharmacists working 5 days per week. For client-related burnout, the logistic regression analysis showed that the pharmacists’ current position in the pharmacy is the independent factor associated with client-related burnout. Staff pharmacists were at higher odds of developing client-related burnout compared to pharmacy managers [(aOR = 2.28: CI = (1.01–5.14), *p* = 0.046)]. The results are presented in Table 4. 

## 4. Discussion 

The study findings showed a high prevalence of burnout among community pharmacists across the three scales of burnout. Personal burnout was noted to be highest, measured on the CBI scale at 83.63%, followed by work-related burnout at 83.19% and client-related burnout at 76.11%. A high prevalence of burnout among community pharmacists was reported across countries in the literature [26,27,28,37,38,57]. A study from Lebanon reported high personal burnout (77.8%), work-related burnout (77.8%), and client-related burnout (89.7%) [28]. A study from Ohio state, USA, reported a burnout prevalence of 67.2% among community-based pharmacist practitioners [38]. A study from Greece reported a high prevalence of burnout among community pharmacists, as 94.3% had high emotional exhaustion, 99.7% had high depersonalization towards service users, and 74.2% lacked feelings of personal accomplishment [27]. These findings are concerning and indicate alarmingly high levels of burnout among community pharmacists, which are higher than burnout levels among other pharmacy sectors [27,35,56]. There are several factors leading to this high level of burnout. These include the demanding nature of the practice in community pharmacies, as they are often the first point of contact for patients and community members due to their convenience and high accessibility, resulting in high patient numbers and numerous free-of-charge consultations in addition to their other responsibilities and duties (i.e., filling prescriptions, counselling, and administrative tasks) [27]. In addition, community pharmacists experience higher levels of work-related stressors, including longer work hours, extra workload, providing administrative tasks and billing related to prescriptions, and negative work–life balance [56]. These are coupled with a lack of resources for burnout [37]. 

In Saudi Arabia, community pharmacists work long hours (>8 h per day) and, typically, 6 days per week. Some community pharmacies operate 24 h a day for 7 days per week [63]. In addition, community pharmacies lack pharmacy technicians to support the pharmacists in their work, leading to pharmacists who must complete all of the tasks and becoming overburdened with administrative and technical tasks [64]. In addition, recently, an e-prescribing service has been introduced, and pharmaceutical services and dispensing of medicines has been transferred from Ministry of Health primary care centers and some hospitals to community pharmacists. This leads to additional workload and challenges encountered by pharmacists in delivering their services, including the need to hire more pharmacy staff [65,66]. In addition, despite the expansion of the roles of community pharmacists and responsibilities, the ratio of community pharmacists per pharmacy remained unchanged in recent years (2.1 pharmacists per community pharmacy in 2022), leading to a higher workload to manage all functions of the pharmacy [44]. Furthermore, despite the long working hours, high workload, and other responsibilities, community pharmacies in Saudi Arabia provide fewer financial incentives and lower salaries and limited opportunities for promotion and development compared to government hospitals, academic institutions, and regulatory bodies, making it the least preferred choice of employment [67,68]. Further, the current practice of community pharmacy in Saudi Arabia is mostly related to dispensing of medicines and supply of health-related products, with limited patient care services leading to less professional satisfaction among community pharmacists. Consequently, all these factors place community pharmacists at a higher risk of burnout. 

The study found that burnout affected community pharmacists regardless of gender. However, it was noted that age and years of experience were associated with burnout among participants; a younger age and fewer years of experience were associated with a higher prevalence of burnout. These findings are consistent with results from other studies which indicated that younger pharmacists and early-career pharmacists are more likely to experience stress and burnout [35,69,70,71]. Consequently, staff pharmacists who were younger were more likely to develop burnout compared to pharmacists in managerial positions in this study. In fact, the challenges of developing a professional identity and adapting to a new work environment can increase the risk of experiencing stress and, ultimately, burnout. Therefore, it is essential to provide mentorship and coaching as support mechanisms to the pharmacists during their early years of practice [72]. In this study, working more than 5 days per week was a risk factor for burnout. Due to the nature of the work of community pharmacists, longer workdays coupled with longer working hours may make pharmacists more prone to increased stress and burnout. Consequently, appropriate working hours per day, an appropriate number of workdays per week, and adequate breaks or days off are crucial to maintain work–life balance. Additionally, our analysis revealed a significant association between the size of the team in community pharmacies and the burnout level. Specifically, community pharmacists who worked in pharmacies with fewer pharmacists had a higher risk of personal burnout. The workload and stress associated with being short-staffed can lead to extended work hours, less flexible schedules, and more frequent patient interactions [36,38].

The study has several implications for practice, policy, and research. The current high prevalence of burnout is concerning and should be urgently addressed. If left unaddressed, it may have detrimental personal and organizational consequences for community pharmacists and their practice. These include high turnover and intentions to leave the community pharmacy for other sectors, reduced productivity, disengagement from the work, and serious mental health issues. All of these may produce a negative impact on pharmacists’ well-being and suboptimal patient care services [56]. For these reasons, many pharmacy organizations have implemented initiatives to address burnout globally. For instance, the American Society of Health-System Pharmacists (ASHP) collaborated with the National Academy of Medicine (NAM) to initiate a well-being and resilience program to reduce burnout and improve patient care and safety [73]. Similarly, the American College of Clinical Pharmacy (ACCP) has called for systemic interventions to address well-being and reduce burnout [74]. Therefore, it is crucial to ensure that burnout and its associated conditions are appropriately addressed among community pharmacists in Saudi Arabia. Initiatives, interventions, and preventive strategies on the individual and organizational levels should be introduced with focus on four key aspects and areas of interventions. First, counselling and awareness programs should be conducted to promote the use of coping strategies such as self-care and individual wellness, including a healthy diet, physical activity/fitness, sufficient sleep, practicing hobbies, emotional health, and encouraging work–life balance [28,38,75]. Second, organizational interventions are essential to proactively address and prevent burnout. Community pharmacy organizations and employers should address the root causes of the work-related stress and promote resilience among pharmacists with organizational or system-level interventions and strategies. These include ensuring adequate pharmacy staff, availability of pharmacy support personnel and technicians, appropriate length of shifts (i.e., working hours per day), the number of workdays per week, and adequate breaks/scheduled breaks [38,76]. In addition to addressing workloads, organizational interventions could include stress management training [27] and ensure that pharmacists have access to resources for resiliency, mentorship, and coaching programs for early-career pharmacists [33]. Third, incentives and recognition programs and rewards are required to increase work engagement and increase satisfaction among employees [33]. Fourth, future research should explore the effectiveness of coping strategies and their impact on pharmacists’ quality of life and well-being. In addition, future studies could explore community pharmacists’ awareness of coping strategies and the level of implementation of these strategies. Furthermore, due to a lack of studies on the current level of training and the extent of coverage of topics on resilience and preventive strategies for burnout in professional practice in the curricula of pharmacy colleges in Saudi Arabia, this topic should be explored by future studies. 

## 5. Strengths and Limitations 

The study provided valuable insights into burnout among community pharmacists and several key factors associated with burnout in Saudi Arabia. The study findings should be the center of attention for policymakers and community pharmacy business owners. However, the study had some limitations. It was conducted among community pharmacists from one region of Saudi Arabia (geographical limitation). Hence, the findings might not be generalizable to the whole country. In addition, convenience sampling was used in this study. This was inevitable as we distributed the survey through in-person field visits and electronically via the WhatsApp application. However, the research team approached community pharmacies across the region, including a wide range of pharmacies and various chain pharmacies. In addition, we recruited a relatively large sample size of community pharmacists. These were carried out to minimize any selection bias and enhance representativeness of the study and generalization of the findings. In addition, participants were assured that their responses were anonymous to minimize any potential bias of socially desirable answers. Another limitation is the cross-sectional nature of the study, which limits the ability to infer causality. However, given these considerations taken to minimize the impact of these factors and given the limited literature in this area, we believe the study will provide future guidance for establishing initiatives and strategies to address the well-being and quality of life among community pharmacists in Saudi Arabia and beyond.

## 6. Conclusions

The study showed that the prevalence of burnout is alarmingly high among community pharmacists in Saudi Arabia. The associated factors with personal burnout included working more than 5 days per week and working in pharmacies with fewer pharmacists. The pharmacists working more than 5 days per week were also at a higher risk of developing work-related burnout. Staff pharmacists were more likely to develop client-related burnout compared to pharmacists in managerial positions in this study. Consequently, it is crucial to address burnout and its associated factors by introducing initiatives, strategies, and interventions that support the well-being, resilience, and quality of life of community pharmacists. These include awareness and support programs, individual-focused interventions, and organizational interventions and strategies to prevent burnout and work-related stress. 

## Figures and Tables

**Table 1:** Participants’ demographic data and work-related characteristics.

Variable	n (%)
Sex	
Male	144 (63.72)
Female	82 (36.28)
Age (in years)	
23–30	151 (66.81)
31–35	43 (19.03)
≥36	32 (14.16)
Experience as a community pharmacist in Saudi Arabia (in years)	
<1 year	76 (33.63)
1–5 years	100 (44.25)
6–10 years	36 (15.93)
>10 years	14 (6.19)
Current position in the pharmacy	
Staff pharmacist	171 (75.66)
Pharmacy manager (in-charge pharmacist)	55 (24.34)
Highest degree or level of education	
Bachelor’s degree	107 (47.35)
PharmD	111 (49.12)
Postgraduate qualification *	8 (3.54)
Current marital status	
Single	139 (61.50)
Married	84 (37.17)
Divorced	1 (0.44)
Widowed	1 (0.44)
Prefer not to say	1 (0.44)
Pharmacist’s number of working hours per day	
<8 h	7 (3.10)
8 h	93 (41.15)
9 h	47 (20.80)
10 h	34 (15.04)
11 h	17 (7.52)
≥12 h	28 (12.39)
Pharmacist’s number of workdays per week	
5 days	24 (10.62)
6 days	143 (63.27)
7 days **	59 (26.11)
Number of pharmacists employed in the pharmacy	
1–2 pharmacists	126 (55.75)
3–4 pharmacists	57 (25.22)
5–6 pharmacists	18 (7.96)
>6 pharmacists	25 (11.06)
Average number of patients and customers served per day	
10–30 patients and customers	26 (11.50)
31–60 patients and customers	70 (30.97)
61–100 patients and customers	66 (29.20)
>100 patients and customer	64 (28.32)
Number of learners/students trained in the pharmacy over the last 12 months	
None	109 (48.23)
1–2 learners	48 (21.24)
≥3 learners	69 (30.53)
Assignment to night shift and duties in the pharmacy in the last 12 months	
Yes	176 (77.88)
No	50 (22.12)

Notes: * Includes postgraduate diploma, master, or doctoral degree, i.e., Ph.D.). ** Includes those who work a mix of 7 days and 6 days per week in a month.

**Table 2:** Prevalence and severity of burnout among the study participants.

Measure and Variables	n (%) *
Personal burnout scale	
Mean (SD)	72.33 ± 22.14
Prevalence (≥50)	189 (83.63)
Level/category	
No/mild (<50)	37 (16.37)
Moderate (50–74)	64 (28.32)
High (75–99)	94 (41.59)
Severe (100)	31 (13.72)
Work-related burnout scale	
Mean (SD)	69.26 ± 21.73
Prevalence (≥50)	188 (83.19)
Level/category	
No/mild (<50)	38 (16.81)
Moderate (50–74)	80 (35.40)
High (75–99)	90 (39.82)
Severe (100)	18 (7.96)
Client-related burnout scale	
Mean (SD)	63.33 ± 25.39
Prevalence (≥50)	172 (76.11)
Level/category	
No/mild (<50)	54 (23.89)
Moderate (50–74)	87 (38.50)
High (75–99)	61 (26.99)
Severe (100)	24 (10.62)

Note: * Unless otherwise stated.

**Table 3:** Association between burnout and participants’ demographic and work-related characteristics.

Variable	n (%)	Personal Burnout		*p* Value *	Work-Related Burnout		*p* Value *	Client-Related Burnout		*p* Value *
		No	Yes		No	Yes		No	Yes	
Sex										
Male	144 (63.72)	27 (18.75)	117 (81.25)	0.20	27 (18.75)	117 (81.25)	0.302	37 (25.69)	107 (74.31)	0.400
Female	82 (36.28)	10 (12.20)	72 (87.80)		11 (13.41)	71 (86.59)		17 (20.73)	65 (79.27)	
Age (in years)										
23–30	151 (66.81)	20 (13.25)	131 (86.75)	**0.044**	19 (12.58)	132 (87.42)	**0.011**	30 (19.87)	121(80.13)	**0.001**
31–35	43 (19.03)	7 (16.28)	36 (83.72)		8 (18.60)	35 (81.40)		8 (18.60)	35 (81.40)	
≥36	32 (14.16)	10 (31.25)	22 (68.75)		11 (34.38)	21 (65.63)		16 (50.00)	16 (50.00)	
Experience as a community pharmacist in Saudi Arabia (in years)										
<1 year	76 (33.63)	11(14.47)	65 (85.53)	0.124	9 (11.84)	67 (88.16)	**0.004**	16 (21.05)	60 (78.95)	**0.006**
1–5 years	100 (44.25)	13 (13.00)	87 (87.00)		12 (12.00)	88 (88.00)		18 (18.00)	82 (82.00)	
6–10 years	36 (15.93)	8 (22.22)	28 (77.78)		12 (33.33)	24 (66.67)		12 (33.33)	24 (66.67)	
>10 years	14 (6.19)	5 (35.71)	9 (64.29)		5 (35.71)	9 (64.29)		8 (57.14)	6 (42.86)	
Current position in the pharmacy										
Staff pharmacist	171 (75.66)	24 (14.04)	147 (85.96)	0.094	22 (12.87)	149 (87.13)	**0.005**	32 (18.71)	139 (81.29)	**0.001**
Pharmacy manager (in-charge pharmacist)	55 (24.34)	13 (23.64)	42 (76.36)		16 (29.09)	39 (70.91)		22 (40.00)	33 (60.00)	
Highest degree										
Bachelor’s degree	107 (47.35)	23 (21.50)	84 (78.50)	0.142	23 (21.50)	84 (78.50)	0.146 ^a^	33 (30.84)	74 (69.16)	**0.006**
PharmD	111 (49.12)	13 (11.71)	98 (88.29)		15 (13.51)	96 (86.49)		17 (15.32)	94 (84.68)	
Postgraduate qualification	8 (3.54)	1 (12.50)	7 (87.50)		0 (0.00)	8 (100.00)		4 (50.00)	4 (50.00)	
Current marital status **										
Single	139 (61.50)	22 (15.83)	117 (84.17)	0.869	19 (13.67)	120 (86.33)	0.196	31 (22.30)	108 (77.70)	0.509
Married	84 (37.17)	14 (16.67)	70 (83.33)		17 (20.24)	67 (79.76)		22 (26.19)	62 (73.81)	
Pharmacist’s number of working hours per day										
<8 h	7 (3.10)	1 (14.29)	6 (85.71)	0.291 ^a^	1 (14.29)	6 (85.71)	0.773 ^a^	2 (28.57)	5 (71.43)	0.609
8 h	93 (41.15)	18 (19.35)	75 (80.65)		15 (16.13)	78 (83.87)		18 (19.35)	75 (80.65)	
9 h	47 (20.80)	5 (10.64)	42 (89.36)		8 (17.02)	39 (82.98)		11 (23.40)	36 (76.60)	
10 h	34 (15.04)	9 (26.47)	25 (73.53)		8 (23.53)	26 (76.47)		12 (35.29)	22 (64.71)	
11 h	17 (7.52)	2 (11.76)	15 (88.24)		1 (5.88)	16 (94.12)		4 (23.53)	13 (76.47)	
≥12 h	28 (12.39)	2 (7.14)	26 (92.86)		5 (17.86)	23 (82.14)		7 (25.00)	21 (75.00)	
Pharmacist’s number of workdays per week										
5 days	24 (10.62)	9 (37.50)	15 (62.50)	**0.011**	10 (41.67)	14 (58.33)	**0.003**	7 (29.17)	17 (70.83)	0.580
6 days	143 (63.27)	21 (14.69)	122 (85.31)		20 (13.99)	123 (86.01)		31 (21.68)	112 (78.32)	
7 days	59 (26.11)	7 (11.86)	52 (88.14)		8 (13.56)	51 (86.44)		16 (27.12)	43 (72.88)	
Number of pharmacists employed in the pharmacy										
1–2 pharmacists	126 (55.75)	14 (11.11)	112 (88.89)	**0.001 ^a^**	15 (11.90)	111 (88.10)	**0.008 ^a^**	31 (24.60)	95 (75.40)	**0.020**
3–4 pharmacists	57 (25.22)	7 (12.28)	50 (87.72)		9 (15.79)	48 (84.21)		8 (14.04)	49 (85.96)	
5–6 pharmacists	18 (7.96)	8 (44.44)	10 (55.56)		8 (44.44)	10 (55.56)		9 (50.00)	9 (50.00)	
>6 pharmacists	25 (11.06)	8 (32.00)	17 (68.00)		6 (24.00)	19 (76.00)		6 (24.00)	19 (76.00)	
Average number of patients and customers served per day										
10–30 patients and customers	26 (11.50)	7 (26.92)	19 (73.08)	0.227	6 (23.08)	20 (76.92)	0.788	7 (26.92)	19 (73.08)	0.134
31–60 patients and customers	70 (30.97)	9 (12.86)	61(87.14)		10 (14.29)	60 (85.71)		23 (32.86)	47 (67.14)	
61–100 patients and customers	66 (29.20)	8 (12.12)	58 (87.88)		11 (16.67)	55 (83.33)		11 (16.67)	55 (83.33)	
>100 patients and customers	64 (28.32)	13 (20.31)	51 (79.69)		11 (17.19)	53 (82.81)		13 (20.31)	51 (79.69)	
Number of learners/students trained in the pharmacy over the last 12 months										
None	109 (48.23)	18 (16.51)	91 (83.49)	0.993	17 (15.60)	92 (84.40)	0.211	26 (23.85)	83 (76.15)	0.302
1–2 learners	48 (21.24)	8 (16.67)	40 (83.33)		12 (25.00)	36 (75.00)		15 (31.25)	33 68.75	
≥3 learners	69 (30.53)	11 (15.94)	58 (84.06)		9 (13.04)	60 (86.96)		13 (18.84)	56 (81.16)	
Assignment to night shift and duties in the pharmacy in the last 12 months										
Yes	176 (77.88)	28 (15.91)	148 (84.09)	0.724	29 (16.48)	147 (83.52)	0.799	42 (23.86)	134 (76.14)	0.984
No	50 (22.12)	9 (18.00)	41 (82.00)		9 (18.00)	41 (82.00)		12 (24.00)	38 (76.00)	

Notes: * Chi-squared test. ** Categories with few numbers (i.e., one participant) were not included in the analysis. ^a^ Fisher’s exact test. Bold indicates statistically significant at *p* < 0.05.

**Table 4:** Associated factors with personal, work-related, and client-related burnout using multivariable logistic regression.

Variable	Adjusted Odds Ratio (AOR)	*p* Value
Associated factors with personal burnout		
Age (in years)		
23–30	2.53 (0.46–13.94)	0.287
31–35	2.42 (0.51–11.610	0.269
≥36	1 (Reference)	
Experience as a community pharmacist in Saudi Arabia (in years)		
<1 year	2.35 (0.29–19.05)	0.423
1–5 years	2.11 (0.30–14.91)	0.453
6–10 years	2.42 (0.43–13.74)	0.319
>10 years	1 (Reference)	
Current position in the pharmacy		
Staff pharmacist	1.31 (0.48–3.63)	0.598
Pharmacy manager (in-charge pharmacist)	1 (Reference)	
Pharmacist’s number of workdays per week		
5 days	1 (Reference)	
6 days	3.60 (1.26–10.27)	**0.017**
7 days	5.15 (1.20–22.20)	**0.028**
Number of pharmacists employed in the pharmacy		
1–2 pharmacists	3.35 (1.06–10.62)	**0.040**
3–4 pharmacists	2.68 (0.76–9.53)	0.127
5–6 pharmacists	0.72 (0.17–3.10)	0.662
>6 pharmacists	1 (Reference)	
Associated factors with work-related burnout		
Age (in years)		
23–30	1.37 (0.23–8.05)	0.726
31–35	2.22 (0.49–10.14)	0.302
≥36	1 (Reference)	
Experience as a community pharmacist in Saudi Arabia (in years)		
<1 year	5.53 (0.59–51.74)	0.134
1–5 years	3.89 (0.50–30.37)	0.196
6–10 years	1.12 (0.21–5.99)	0.892
>10 years	1 (Reference)	
Current position in the pharmacy		
Staff pharmacist	1.70 (0.65–4.43)	0.279
Pharmacy manager (in-charge pharmacist)	1 (Reference)	
Pharmacist’s number of workdays per week		
5 days	1 (Reference)	
6 days	5.78 (1.96–17.06)	**0.001**
7 days	8.38 (1.99–35.27)	**0.004**
Number of pharmacists employed in the pharmacy		
1–2 pharmacists	2.03 (0.56–7.35)	0.279
3–4 pharmacists	1.15 (0.30–4.41)	0.840
5–6 pharmacists	0.741 (0.15–3.67)	0.714
>6 pharmacists	1 (Reference)	
Associated factors with client-related burnout		
Age (in years)		
23–30	0.91 (0.20–4.08)	0.904
31–35	1.62 (0.40–6.63)	0.501
≥36	1 (Reference)	
Experience as a community pharmacist in Saudi Arabia (in years)		
<1 year	2.12 (0.31–14.30)	0.442
1–5 years	2.64 (0.43–16.30)	0.296
6–10 years	2.15 (0.43–10.85)	0.355
>10 years	1 (Reference)	
Current position in the pharmacy		
Staff pharmacist	2.28 (1.01–5.14)	**0.046**
Pharmacy manager (in-charge pharmacist)	1 (Reference)	
Highest degree or level of education		
Bachelor’s degree	1.90 (0.35–10.26)	0.457
PharmD	4.08 (0.69–24.02)	0.120
Postgraduate qualification (postgraduate diploma, master’s, or doctoral degree, i.e., Ph.D.)	1 (Reference)	
Number of pharmacists employed in the pharmacy		
1–2 pharmacists	1.42 (0.48–4.20)	0.524
3–4 pharmacists	2.44 (0.69–8.63)	0.168
5–6 pharmacists	0.44 (0.10–1.87)	0.266
>6 pharmacists	1 (Reference)	

Note: Bold indicates statistically significant at *p* < 0.05.

## Data Availability

Additional data are available upon reasonable request from the corresponding author.

## Supplementary Materials

The following supporting information can be downloaded at: https://www.mdpi.com/article/10.3390/healthcare12181834/s1. Table S1: Univariable logistic regression analysis of factors associated with personal burnout. Table S2. Univariable logistic regression analysis of factors associated with work-related burnout. Table S3. Univariable logistic regression analysis of factors associated with client-related burnout.
