# Supplementary material for: Prevalence and Factors Associated with Burnout among Community Pharmacists in Saudi Arabia: Findings and Implications
Source: Healthcare (Basel). 2024 Sep 13;12(18):1834. doi: 10.3390/healthcare12181834 (PMC11430934; doi:10.3390/healthcare12181834)
Supplement: Supplementary file 1 [file healthcare-12-01834-s001.zip › healthcare-3150770-supplementary.pdf]

Supplementary Tables: Univariate logistic regression analysis

Table S1. Univariate logistic regression analysis of factors associated with personal burnout.

Variable	Crude Odds Ratio (COR)	*p* Value
**Sex**		
Male	0.60 (0.28–1.32)	0.204
Female	1	
**Age (in years)**		
23–30	2.98 (1.23–7.20)	**0.015**
31–35	2.34 (0.78–7.04)	0.131
≥36	1	
**Experience as a community pharmacist in Saudi Arabia (in years)**		
<1 year	3.28 (0.93–11.65)	0.066
1–5 years	3.72 (1.08–12.83)	**0.038**
6–10 years	1.94 (0.51–7.47)	0.333
>10 years	1	
**Current position in the pharmacy**		
Staff pharmacist	1.896 (0.89–4.04)	0.098
Pharmacy manager (in–charge pharmacist)	1	
**Highest degree or level of education**		
Bachelor's degree	0.52 (0.06–4.46)	0.552
PharmD	1.08 (0.12–9.47)	0.947
Postgraduate qualification (postgraduate diploma, master's, or doctoral degree, i.e., Ph.D.)	1	
**Current marital status ***		
Single	1.06 (0.51–2.21)	0.869
Married	1	
**Pharmacist's number of working hours per day**		
<8 hr	1	
8 h	0.69 (0.08–6.14)	0.743
9 h	1.40 (0.14–14.12)	0.775
10 h	0.46 (0.05–4.39)	0.502
11 h	1.25 (0.10–16.50)	0.865
≥12 h	2.17 (0.17–28.01)	0.554
**Pharmacist's number of workdays per week**		
5 days	1	
6 days	3.49 (1.35–8.99)	**0.010**
7 days	4.46 (1.42–13.97)	**0.010**
**Number of pharmacists employed in the pharmacy**		
1–2 pharmacists	3.77 (1.38–10.31)	**0.010**
3–4 pharmacists	3.36 (1.06–10.66)	**0.039**
5–6 pharmacists	0.59 (0.17–2.06)	0.407
>6 pharmacists	1	
**Average number of patients and customers served per day**		
10–30 patients and customers	0.69 (0.24–1.10)	0.495
31–60 patients and customers	1.73 (0.68–4.37)	0.248
61–100 patients and customers	1.85 (0.71–4.82)	0.209
>100 patients and customer	1	
**Number of learners/students trained in the pharmacy over the last 12 months**		

None	1	
1–2 learners	0.96 (0.42–2.18)	0.920
≥3 learners	0.95 (0.35–2.57)	0.917
**Assignment to night shift and duties in the pharmacy in the last 12 months**		
Yes	1.16 (0.51–2.65)	0.725
No	1	

Note: * Categories with few numbers (i.e., one participant) were not included in the analysis.

Table S2. Univariate logistic regression analysis of factors associated with work-related burnout.

**Variable**	**Crude Odds Ratio (COR)**	***p* Value**
**Sex**		
Male	0.67 (0.31–1.44)	0.305
Female	1	
**Age (in years)**		
23–30	3.64 (1.52–8.72)	**0.004**
31–35	2.29 (0.80–6.61)	0.125
≥36	1	
**Experience as a community pharmacist in Saudi Arabia (in years)**		
<1 year	4.14 (1.13–15.11)	**0.032**
1–5 years	4.07 (1.17–14.20)	**0.027**
6–10 years	1.11 (0.31–4.05)	0.873
>10 years	1	
**Current position in the pharmacy**		
Staff pharmacist	2.78 (1.33–5.79)	**0.006**
Pharmacy manager (in–charge pharmacist)	1	
**Highest degree or level of education**		
Bachelor's degree		
PharmD		
Postgraduate qualification (postgraduate diploma, master's, or doctoral degree, i.e., Ph.D.)		
**Current marital status ***		
Single	1.60 (0.78–3.29)	0.20
Married	1	
**Pharmacist's number of working hours per day**		
<8 h	1	
8 h	0.87 (0.10–7.73)	0.898
9 h	0.81 (0.09–7.71)	0.856
10 h	0.54 (0.06–5.19)	0.595
11 h	2.67 (0.14–49.76)	0.511
≥12 h	0.77 (0.08–7.86)	0.823
**Pharmacist's number of workdays per week**		
5 days	1	
6 days	4.39 (1.72–11.23)	**0.002**
7 days	4.55 (1.51–13.71)	**0.007**
**Number of pharmacists employed in the pharmacy**		
1–2 pharmacists	2.34 (0.81–6.78)	0.118
3–4 pharmacists	1.68 (0.53–5.38)	0.379
5–6 pharmacists	0.40 (0.11–1.46)	0.163
>6 pharmacists	1	
**Average number of patients and customers served per day**		

10–30 patients and customers	0.69 (0.23–2.12)	0.519
31–60 patients and customers	1.25 (0.49–3.17)	0.645
61–100 patients and customers	1.04 (0.42–2.60)	0.937
>100 patients and customer	1	
**Number of learners/students trained in the pharmacy over the last 12 months**		
None	1	
1–2 learners	0.81 (0.34–1.94)	0.639
≥3 learners	0.45 (0.17–1.17)	0.102
**Assignment to night shift and duties in the pharmacy in the last 12 months**		
Yes	1.11 (0.49–2.54)	0.80
No	1	

Note: * Categories with few numbers (i.e., one participant) were not included in the analysis.

Table S3. Univariate logistic regression analysis of factors associated with client-related burnout.

**Variable**	**Crude Odds Ratio (COR)**	***p* Value**
**Sex**		
Male	0.76 (0.39–1.45)	0.401
Female	1	
**Age (in years)**		
23–30	4.03 (1.81–8.98)	**0.001**
31–35	4.38 (1.56–12.31)	**0.005**
≥36	1	
**Experience as a community pharmacist in Saudi Arabia (in years)**		
<1 year	5.00 (1.52–16.49)	**0.008**
1–5 years	6.07 (1.88–19.67)	**0.003**
6–10 years	2.67 (0.75–9.45)	**0.129**
>10 years	1	
**Current position in the pharmacy**		
Staff pharmacist	2.90 (1.49–5.62)	**0.002**
Pharmacy manager (in–charge pharmacist)	1	
**Highest degree or level of education**		
Bachelor's Degree	2.24 (0.53–9.52)	**0.273**
PharmD	5.53 (1.26–24.27)	**0.023**
Postgraduate Qualification (postgraduate diploma master's or doctoral degree, i.e., Ph.D.)	1	
**Current marital status ***		
Single	1.24 (0.66–2.32)	0.509
Married	1	
**Pharmacist's number of working hours per day**		
<8 h	1	
8 h	1.67 (0.30–9.30)	0.560
9 h	1.31 (0.22–7.71)	0.766
10 h	0.73 (0.12–4.37)	0.733
11 h	1.30 (0.18–9.47)	0.796
≥12 h	1.20 (0.19–7.63)	0.847
**Pharmacist's number of workdays per week**		
5 days	1	
6 days	1.49 (0.57–3.91)	0.420
7 days	1.11 (0.39–3.17)	0.850

**Number of pharmacists employed in the pharmacy**		
1–2 pharmacists	0.97 (0.36–2.64)	0.949
3–4 pharmacists	1.93 (0.59–6.32)	0.275
5–6 pharmacists	0.32 (0.09–1.16)	0.083
>6 pharmacists	1	
**Average number of patients and customers served per day**		
10–30 patients and customers	0.69 (0.24–1.10)	0.495
31–60 patients and customers	0.52 (0.24–1.14)	0.104
61–100 patients and customers	1.28 (0.52–3.10)	0.593
>100 patients and customer	1	
**Number of learners/students trained in the pharmacy over the last 12 months**		
None	1	
1–2 learners	0.74 (0.35–1.56)	0.432
≥3 learners	0.51 (0.22–1.21)	0.125
**Assignment to night shift and duties in the pharmacy in the last 12 months**		
Yes	1.00 (0.48–2.10)	0.984
No	1	

Note: * Categories with few numbers (i.e., one participant) were not included in the analysis.
